# Biomarkers for ideal protein: rabbit diet metabolomics varying key amino acids

**DOI:** 10.1038/s42003-024-06322-2

**Published:** 2024-06-10

**Authors:** Pablo Jesús Marín-García, Lola Llobat, María Cambra-López, Enrique Blas, Torben Larsen, Juan José Pascual, Mette Skou Hedemann

**Affiliations:** 1 Department of Animal Production and Health, Veterinary Public Health and Food Science and Technology (PASAPTA), Institute of Biomedical Sciences, Cardenal Herrera-CEU University, CEU Universities, Valencia, Spain; 2 Institute for Animal Science and Technology, Universitat Politècnica de València, Valencia, Spain; 3 Department of Animal and Veterinary Sciences, Aarhus University, Tjele, Denmark

**Keywords:** Animal physiology, Metabolomics

## Abstract

With the main aim of identifying biomarkers that contribute to defining the concept of ideal protein in growing rabbits under the most diverse conditions possible this work describes two different experiments. Experiment 1: 24 growing rabbits are included at 56 days of age. The rabbits are fed *ad libitum* one of the two experimental diets only differing in lysine levels. Experiment 2: 53 growing rabbits are included at 46 days of age, under a fasting and eating one of the five experimental diets, with identical chemical composition except for the three typically limiting amino acids (being fed commercial diets *ad libitum* in both experiments). Blood samples are taken for targeted and untargeted metabolomics analysis. Here we show that the metabolic phenotype undergoes alterations when animals experience a rapid dietary shift in the amino acid levels. While some of the differential metabolites can be attributed directly to changes in specific amino acids, creatinine, urea, hydroxypropionic acid and hydroxyoctadecadienoic acid are suggested as a biomarker of amino acid imbalances in growing rabbits’ diets, since its changes are not attributable to a single amino acid. The fluctuations in their levels suggest intricate amino acid interactions. Consequently, we propose these metabolites as promising biomarkers for further research into the concept of the ideal protein using rabbit as a model.

## Abstract (web summary)

A metabolomics study, using rabbits as a model, indicates the existence of metabolomic profiles that can enhance protein utilization in diets.

## Introduction

The concept of the “ideal protein” encompasses a specific combination of amino acids that optimises protein utilisation^1^, leading to maximal retention^2^ and minimal excretion^3^. Achieving this ideal protein formulation requires a comprehensive understanding of the precise nutritional requirements tailored to each specific animal, aligning with the dynamic nature of precision nutrition^4^. In a context where dietary protein recommendation for rabbits has been reduced^5^, while fibre content has been increased^6^, understanding the precise proportion of amino acids required for animals is particularly interesting.

A diet with an amino acid imbalance leads animals to catabolise the remaining amino acids, incurring an energetic cost for the animal and resulting in the expenditure of ATP molecules proportionally with the nitrogen excreted^7^. In essence, precise adjustment of amino acid content not only enhances productivity and improved animal health, thereby enhancing overall animal welfare, but also contributes to reduced environmental pollution.

As previously described, the ideal protein concept aligns with precision nutrition. In this framework, untargeted metabolomics emerges as a powerful tool to investigate the metabolome of individuals and is proving valuable in livestock production^8,9^, including effects on amino acid nutrition^10^. One of the most interesting application of untargeted metabolomics is to find biomarkers^11^. Searching for biomarkers, defined as objectively measurable characteristics^12^, has already been used to study dietary patterns^13^ or dietary composition^14^. In this study, we aimed to obtain biomarkers that could refine the understanding of the ideal protein concept.

A deficiency in essential amino acids can render them limiting factors, resulting in an unbalanced diet. In the case of rabbits, lysine, sulfur amino acids and threonine are the most typical limiting amino acids^15–17^. The requirements are well established and the dietary levels are determined as 8.1, 5.8 and 6.9 g/kg DM for lysine, sulfur amino acids and threonine, respectively^18^, appointed as the baseline levels in this work.

Growing rabbits are characterised by having many small daily meals, unlike other species eating only in 1 or 2 daily meals^19–21^. The broad range of possible diets combinations, with explained above, causes great potential variability in results. Hence, this study concentrates on two distinct experiments to derive extrapolated conclusions, utilising the rabbit as a model.

This work hypothesises that short-term changes in the dietary levels of three frequently limiting amino acids – lysine, methionine and threonine - in growing rabbits will influence the animals’ metabolic phenotype. These changes of metabolic phenotypes have already been used to improve the existing methods for estimating amino acid requirements in other species^22^ and could be used as a tool for the conservation of this species in the wild^23,24^. The objective is to confirm these alterations and pinpoint possible specific biomarkers signalling an excess or deficiency in a particular amino acid. Moreover, the study aims to uncover biomarkers to evaluate protein in diets (imbalances), moving toward a better understanding of the concept of an ideal protein.

## Results

### Experiment 1

**Fig. 1:**
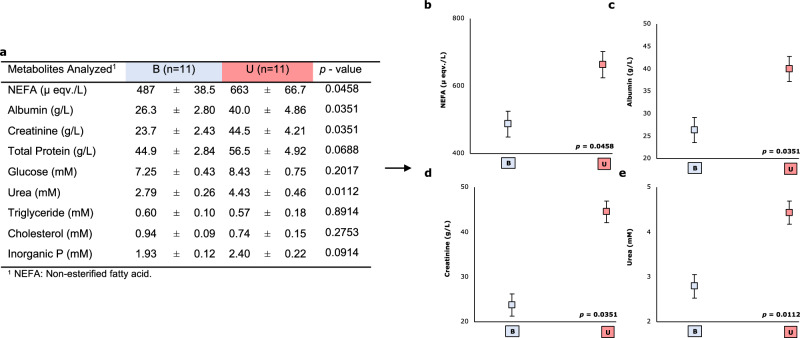
Summary of the results obtained by the targeted metabolomics test of the Experiment 1. Effects of the experimental diet (B: Balanced diet; U: Unbalanced diet) on the results obtained by targeted metabolomics. **a** Comparison between experimental diets of all metabolites analysed by targeted metabolomics. Effects of the physiological status on non-esterified fatty acid **b**, albumin **c**, creatinine **d** and urea **e** measured in growing rabbit plasma (*n* = 24). LS means and standard error (error bars).

Daily Feed intake (on av. 181 g/d) and daily weight gain (on av. 53 g/d) was recorded to ensure the health of the animals. Figure 1 represents the effects of the experimental diet on the targeted metabolomics analysis. Targeted metabolomics showed that animals fed with the unbalanced diet (Diet U) showed higher NEFA (+36%; *p* = 0.0458), albumin (+52%; *p* = 0.0351), creatinine (+88%; *p* = 0.0351), urea (+59%; *p* = 0.0112) and a trend in total protein (+26%; *p* = 0.00688) levels than animals fed with the balanced diet (Diet B). Nevertheless, experimental diet did not affect glucose, triglyceride, cholesterol or inorganic P.

**Fig. 2:**
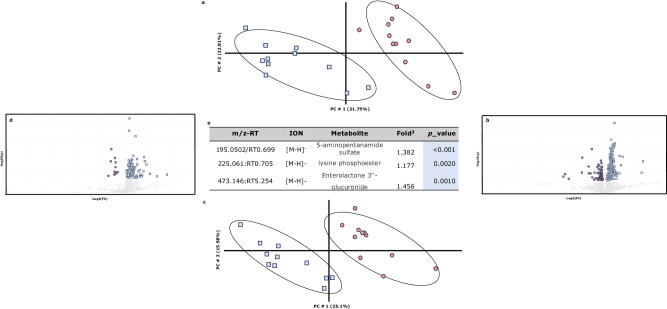
Summary of the results obtained by the untargeted metabolomics test of the Experiment 1. PLS-DA score plot of the plasma metabolome in positive (*R*^2^ =  0.9600 *Q*^2^ = 0.6290) (**a**) and negative mode (*R*^2^ = 0.9420 *Q*^2^ = 0.6800) (**c**), respectively. The colours correspond to the two experimental diets:  Animals fed Diet B and  : Animals fed Diet U. **b**, **d** Volcano plot showing differentially significant metabolites between two experimental diets (Two-sided Wilcoxon rank tests with the value adjusted by false discovery rate, FDR <  0.05) are shown;  (Fold change > 1.234)  (fold change < 0.95) in the volcano plot. Volcano plots are in positive and negative mode, respectively (*r* = 0.5765 and *r* = 0.6808, respectively). **e** List of plasmatic metabolites discriminating between experimental diets (Diet B vs. Diet U). Metabolites were tentatively identified using MS/MS and METLIN/HMDB databases. m/z-RT corresponds to query masses and retention time.

Figure 2 represents the results obtained by untargeted metabolomics. Figure 2a, c represent the first two principal components obtained by PLS-DA of untargeted metabolomics data in positive and negative mode, respectively. As shown, the variability associated with these principal components obtained from the metabolic profile (45% and 41%, respectively of the total) can be used to differentiate the experimental diets, as there is no overlap between groups. In the volcano graphs (Fig. 2b, d for positive and negative mode, respectively), the metabolites responsible for the discrimination between the experimental diets could be observed. After the identification, Fig. 2e summarises the tentatively identified metabolites that explain the differences between groups. Animals fed Diet B showed higher plasmatic levels of 5-aminopentanamide (+38%; *p* < 0.001), lysine phosphoester (+18%; *p* = 0.0020) and enterolactone 3″-glucuronide (+46%; *p* = 0.0010) than animals fed Diet U.

### Experiment 2

**Fig. 3:**
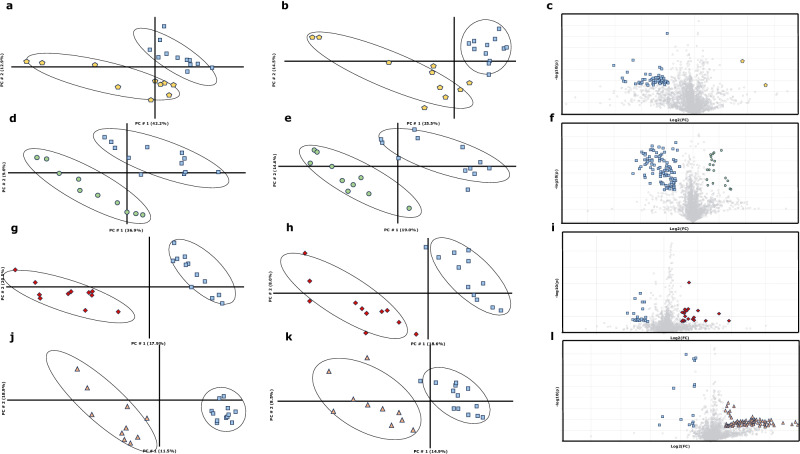
Summary of the results obtained by the untargeted metabolomics test of the Experiment 2. **a**, **b**,**d**, **e**, **g**, **h**,**j**,**k** Partial Least Squares models of plasma in positive (left) and negative mode (right) for different experimental diets compared with the reference levels.  Diet HHH,  Diet LLL,  Diet LMM,  Diet MHL and  Diet MMM. (*R*^2^ = 0.9968, *Q*^2^ = 0.3913; *R*^2^ = 0.9560, *Q*^2^ = 0.2930; *R*^2^ = 0.9450, *Q*^2^ = 0.3880; *R*^2^ = 0.9983, *Q*^2^ = 0.298^2^ ; *R*^2^ = 0.9999, *Q*^2^ = 0.7830; *R*^2^ = 0.9546, *Q*^2^ = 0.6336; *R*^2^ = 0.9760, *Q*^2^ = 0.5880; *R*^2^ = 0.9330, *Q*^2^ = 0.5420 for **a**, **b**, **d**, **e**, **g**, **h**, **j**, **k**, respectively) **c**, **f**, **i**, **l** Volcano plot displaying differentially abundant metabolites between the experimental diets. Two-sided Wilcoxon rank tests with the value adjusted by false discovery rate (FDR < 0.05) are shown. Each icon refers to the highest or lowest levels in the reflected experimental diet.

A summary of the results obtained by untargeted metabolomics is shown in Fig. 3. Figure 3a–k represent the first two principal components obtained by PLS-DA of untargeted metabolomics data in positive (left side) and negative (right side) mode, respectively. As shown, the variability associated with these principal components obtained from the metabolic profile (55%, 40%, 47%, 34%, 38%, 27%, 30% and 23%, respectively of the total) can be used to differentiate experimental diets, as there is no overlap between diets. In the volcano plots (Fig. 3c, f, i, l), the metabolites responsible for the discrimination between the experimental diets could be observed. In Table 1, the tentatively identified metabolites discriminating between experimental diets are listed. In summary, diets with different levels of lysine, sulfur amino acids and threonine showed distinct levels of 3-methyl sulfolene (*p* < 0.001), methionine (*p* < 0.001), linoleamide (*p* = 0.0005), hydroxypropionic acid (*p* < 0.001), hydroxyoctadecadienoic acid (*p* = 0.0024), dodecyl sulfate (*p* = 0.0026), citric acid (*p* = 0.0093), isocitric acid (*p* < 0.0233), bile acid (*p* = 0.0973) and 3,4-dihydroxyphenylvaleric acid (*p* = 0.0129).

**Table 1:** Identification of discriminating metabolites between experimental diets (n = 53)

m/z-RT^a^	Ion	Metabolite	*p* - value
133.0314RT1.147	[M + H]+	3-Methyl sulfolene	<0.001
150.0583RT1.147	[M + H]+	Methionine	<0.001
280.26309RT11.601	[M + H]+	Linoleamide	0.0005
89.024RT1.087	[M-H]-	Hydroxypropionic acid	<0.001
295.22791RT9.995	[M-H]-	Hydroxyoctadecadienoic acid	0.0024
265.1474RT8.979	[M-H]-	Dodecyl sulfate	0.0026
191.019RT1.263	[M-H]-	Citric acid	0.0093
191.0195RT0.889	[M-H]-	Isocitric acid	0.0233
448.306RT7.289	[M-H]-	Bile acid (Formula)	0.0973
209.079RT5.074	[M-H]-	3,4-Dihydroxyphenylvaleric acid	0.0129

^a^Identification was done tentatively identified using MS/MS and METLIN/HMDB databases.

**Fig. 4:**
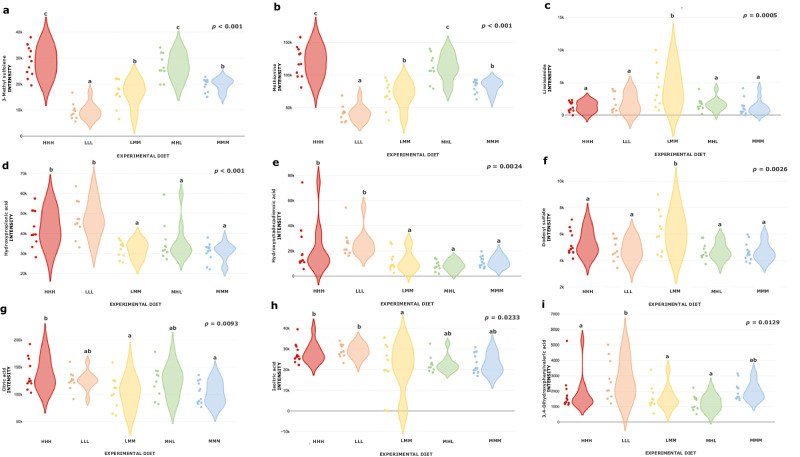
Violin plots showing the intensity of discriminating metabolites of the Experiment 2. **a** 3-Methyl sulfolene, **b** Methionine, **c** Linoleamide, **d** Hydroxypropionic acid, **e** Hydroxyoctadecadienoic acid, **f** Dodecyl sulphate, **g** Citric acid, **h** Isocitric acid, **i** 3,4-dihydroxyphenylvaleric acid as function of the experimental diet  Diet HHH,  Diet LLL,  Diet LMM,  Diet MHL and  Diet MMM. **a**–**c** Least square means in the graph with no common superscripts differ significantly at *p* < 0.05.

Figure 4 summarises the violin plots illustrating the intensity of identified metabolites that elucidate the distinctions between experimental diets. Comparing with a diet formulated according to current recommendations (Diet MMM), Diet HHH showed higher levels of 3-methyl sulfolene (+45%, *p* = 0.0002), methionine (+44%, *p* < 0.001), hydroxypropionic acid (+39%, *p* = 0.0044), hydroxyoctadecadienoic acid (+91%, *p* = 0.0300) and citric acid (+34%, *p* = 0.0173). Diet LLL showed higher levels of hydroxypropionic acid (+53%, *p* < 0.001), hydroxyoctadecadienoic acid (+130%, *p* = 0.0035) and lower levels of 3-methyl sulfolene (−48%, *p* < 0.001) and methionine (−50%, *p* < 0.001) than Diet MMM. Diet LMM showed higher levels of dodecyl sulfate (+33%, *p* = 0.0040) than Diet MMM. Finally, Diet MHL showed higher levels of 3-methyl sulfolene (+36%, *p* = 0.0046) and methionine (+34%, *p* = 0.0041) than Diet MMM. In terms of validation, the average *R*^2^ and *Q*^2^ obtained from the two experiments were 0.96 and 0.57, respectively.

## Discussion

### Experiment 1

Application of untargeted metabolomics to rabbit studies has increased recently and it has been used for various purposes^25–27^. However, to our knowledge, no works exploring the plasma metabolome of growing rabbits fed on a diet with the presence of some limiting amino acid have been published (lysine in this case). Thus, the aim of this work was to find possible biomarkers (measured by targeted and untargeted metabolomics) of lysine as limiting amino acid.

Regarding the targeted metabolomics analyses, animals fed on Diet U exhibited elevated levels of NEFA, albumin, creatinine and urea. Each of these components is expounded upon below. NEFA undergo metabolism through beta-oxidation at the mitochondrial level to generate energy. The NEFA levels, averaging 575 µ eqv./L in this study, align closely with values reported for productive rabbits^28,29^, and are lower than those noted in the same wild species^30–32^. In this work, we have obtained 36% more of this metabolite in animals fed on Diet U. While NEFA is typically associated with energy metabolism, in this trial both diets are isoenergetic at the digestible level. Consequently, the observed differences are likely attributable to protein quality. A plausible explanation is that the unbalanced amino acid profile of Diet U could induce a shift away from protein as the primary energy source, accounting for the elevated NEFA levels, in this context we have a diet with moderate levels of crude protein but, in Diet U, an amino acid profile formulated to produce an imbalance, which should cause a protein limitation. This hypothesis gains support from similar findings in other productive animals, such as dairy cows, where increased NEFA levels resulted from amino acid deficiencies in protein-restricted diets^33^, and growing pigs, where the levels of amino acids in protein-restricted diets modulated the energy metabolism^34^.

Albumin, the main protein in plasma, plays a key role in modulating plasma oncotic pressure^35^. Previous analyses of albumin levels in productive and wild rabbits align with the data obtained in this study^36^. The potential role of amino acid nutrition in serum albumin has been studied^37^. In fact it has been demonstrated that albumin levels could be associated with the quality of dietary protein^38^. In this work, albumin levels were 52% higher in Diet U.

In this work, creatinine levels were 88% higher in Diet U. Creatinine is an amino acid derivative. Creatinine is derived from glycine, l-arginine and S-adenosyl-l-methionine^39^. It has different functions in the protein and energy metabolism^40^, and it is found in arginine and proline metabolism^41^. Despite limited studies on creatinine measurements in rabbits, the levels obtained in this work were notably higher than those reported by Onifade et al.^42^, possibly due to differences in measurement (plasma vs. serum)^42^. Creatinine is crucial for energy transfer in vertebrate cells^43^ and a relationship between this metabolite and the quality of the diet has been demonstrated.

After feeding Diet U a higher urea level was observed. Urea is the result from ammonia produced by the deamination of amino acids. Urea nitrogen level is the main result of urine excretion and has been widely used to detect amino acid imbalances in animal diets^44^. Urea levels have previously been analysed in growing rabbits, and the levels found in this work are similar to those obtained previously^2,45,46^. It has been demonstrated that urea levels could be associated with the presence of some limiting amino acid^38^.

The three metabolites (albumin, creatinine and urea) exhibit interrelated patterns^47,48^. The higher levels of these three metabolites could be attributed to the lysine deficit of Diet U. This imbalance leads to a greater number of amino acids remaining unused by the animal, increasing their excretion. On the contrary, animals fed on Diet B show low urea levels in the blood, indicating a decrease in protein catabolism and more efficient total N utilisation^49,50^. Amino acids absorbed but not used (in this trial, we have the same quantity absorbed in each of the diets, but with a clear limiting amino acid in Diet U), together with the ones coming from cell renewal, are catabolised in the urea cycle, increasing the urea levels. The urea cycle also plays a role in creatinine synthesis^51^. Furthermore, albumin synthesis is stimulated by those amino acids which increase urea synthesis^52^. In summary, in Diet U there would be more substrate to generate these metabolites, proportionally increasing their levels in the animals fed the unbalanced feed. Any of them can be used as a possible biomarker. These are the metabolites that exhibited variations in our targeted metabolomics analyses. However, it’s essential to note that our quantification efforts were focused solely on nine metabolites.

Concerning untargeted metabolomics, higher levels of 5-aminopentanamide, lysine phosphoester and enterolactone 3′- glucoronide were identified in animals fed on Diet B. Each of them is described in more detail below.

5-aminopentanamide is involved in the lysine degradation IV pathway and can be produced through the enzymatic reduction of 5-aminopentanoate or enzymatic oxidation of l-lysine^53^. In this work, we obtained 38% more of this metabolite in animals fed on Diet B. Lysine phosphoester belongs to the class of organic compounds known as alpha amino acids and derivatives. Lysine phosphoester arises as a result of protein phosphorylation (which involves the addition of a phosphoryl group)^54^. This protein phosphorylation has recently been described in lysine^55^ as a result of lysine degradation. In this work, we obtained 18% more of this metabolite in animals fed on Diet B than in animals fed Diet U. Both metabolites, linked to lysine degradation, suggest a higher lysine degradation in Diet B. This increased degradation could imply the utilisation of lysine for meeting the animal’s energy requirements through post-absorptive oxidation to generate energy^56^, or that there is more lysine than needed and hence the surplus is degraded/oxidised.

This greater degradation of lysine could be explained with different hypotheses: (i) proportional issue; the higher availability of lysine in Diet B may lead to a proportional increase in its degradation. This is supported by the fact that dietary lysine levels in Diet B were 84% higher than in Diet U. (ii) Oversized levels: Another hypothesis suggests that the levels of this amino acid are oversized, causing an excess that must be catabolised. This hypothesis seems to be less plausible, as there are recent works in growing rabbits of this same age and selected for similar traits that showed that the lysine levels used in this experiment match their requirements^57,58^. (iii) Higher digestibility of synthetic lysine: The increased degradation might be a consequence of the higher digestibility of the synthetic lysine added to Diet B by adding l-lysine HCl (4.7 g per kg). This greater digestibility and characteristics of synthetic amino acids may mean that absorption is not simultaneous, generating a greater initial amount of lysine to be degraded^16,17,59^. Based on literature search, this is the first time that 5-aminopentanamide, lysine phosphoester and enterolactone 3′ sulfate have been reported in growing rabbits.

In general terms, we have observed 56% more enterolactone 3′- glucoronide in animals fed with an unbalanced diet -with lower lysine levels. Enterolactone is related to different aspects of animal physiology: (i) changes in gut microbiome, quite unlikely in this experiment due to the short exposure to the experimental diets. This is the case were it has been sown low levels of enterolactone are related to changes in gut bacteria^60^ and associated with protective effect on liver function^61^. (ii) The conversion of lignin into mammalian lignans (e.g. enterolactone) has previously been demonstrated in rats^62^. Enterolactone is formed by the action of intestinal bacteria on plant lignin precursors present in the diet^63^. The diet of rabbit is rich in lignin and a reduction in the plasma level of the enterolactone could be a marker of a low lignin intake. In this trial, lignin dietary concentration is not affected, but there is a tendency to greater intake of Diet B.

### Experiment 2

This Experiment demonstrates that short-term diet changes have an impact on the metabolome due to different amino acids dietary content. These rapid changes have already been observed in other species as humans^64^, pigs^65,66^ and birds^67^, but this is the first time that it has been shown in growing rabbits. The effort in this work is dedicated to enhancing the assessment of dietary amino acid intake with the main aim of identifying one or more meaningful biomarkers^68^.

3-Methyl sulfolene belongs to fragment of methionine amino acids. Experimental diets with elevated methionine levels (Diets MHL and HHH) showed the highest intensities for both metabolites. Conversely, diets with average methionine levels (Diets LMM and MMM) displayed intermediate levels. Finally, diet with the lowest methionine level (Diet LLL) showed the lowest levels of these plasmatic metabolites. This suggests a catabolism of methionine which indicates that it is not being fully used by the animals. This methionine degradation could be explained through different hypotheses: (i) Proportional Issue: It is possible that the higher availability of methionine leads to a proportional increase in its degradation. This is supported by different methionine content levels in experimental diets (H: 4.23 g/kg DM; M: 3.43 g/kg DM; L: 2.53 g/kg DM). (ii) Oversized Levels: Another hypothesis suggests that the levels of this amino acid are oversized, resulting in an excess that must be catabolised. However, this hypothesis seems less plausible, as the nutritional requirements for methionine are well established and closer to the higher levels^58^. (iii) Higher Digestibility of Synthetic Methionine: The increased degradation might be a consequence of the higher digestibility of the synthetic methionine added at each level. The improved digestibility and characteristics of synthetic amino acids may lead to non-simultaneous absorption, generating a greater initial amount of methionine to be degraded^16,17,59,69,70^.

Citric acid has been used as biomarker of physiological response to diet^71^. This metabolite is crucial in different metabolic pathways related with amino acids^72^ and it has been demonstrated to involve the final pathway for protein degradation^73^. In fact, it has been observed that citric acid could be related to protein metabolism^74^ and urea cycle^75^. In this work, Diet HHH showed higher citric acid levels than Diet MMM, which could be explained by the increase in unnecessary amino acids that would increase the degradation and amino acid elimination. In a previous trial, it was found that animals with high growth potential did not choose Diet HHH when it was offered with the Diet MMM throughout the growing period, which suggests that they do not have higher amino acid requirements^57^. In the cases where amino acid catabolism occurs (in this case due to an oversupply), an increase in acetyl-CoA also occurs (acetyl-CoA is an anaplerotic molecule of the citric acid cycle)^76^. Because of these factors, an amino acid oversupply is expected to result in elevated acetyl-CoA production, consequently increasing the concentrations of intermediate metabolites in the citric acid cycle, including citric acid itself. This phenomenon would account for its heightened presence in blood samples^77^.

Changes in the concentration of a specific metabolite within diets sharing identical levels of a particular amino acid cannot be attributed to variations in the concentration of the cited amino acid. This holds true for metabolites such as hydroxypropionic acid and hydroxyoctadecadienoic acid. The fluctuations in these metabolites cannot be elucidated by direct individual assessments of lysine levels, as evidenced by the dissimilar levels of this metabolite in Diet LLL and Diet LMM. Similarly, the levels of sulfur amino acids do not provide a singular explanation, given the disparate concentrations in Diet HHH and Diet MHL. Finally, threonine levels alone cannot account for the observed differences, exemplified by the varying concentrations in Diet LLL and Diet MHL. The only plausible explanation for different levels of hydroxypropionic acid and hydroxyoctadecadienoic acid lies in the complex interactions among the amino acids comprising the diet. In this case, it is observed that the trends between the diets are the same for both metabolites, being higher in Diets HHH and LLL, and lower in diets LMM, MHL and MMM.

Hydroxyoctadecadienoic is a stable oxidation product of linoleic acid^78^. The influence of linoleic acid on flux of metabolites in growing pigs has been shown^79^, which is interesting in relation to this work where only the amino acids levels differ. On the other hand, hydroxypropionic acid is a carboxylic acid and serves as an intermediate in the breakdown of branched-chain amino acids (BCAAs). BCAAs include leucine, isoleucine and valine^80^. Other hydroxy acids in urine have been shown to be related with products from BCAAs degradation from ketogenesis^81^ and excretion pattern of BCAAs^82^. The levels in our experimental diets of leucine, isoleucine and valine are identical (9.70, 5.07 and 7.03 respectively) so, in this case, an increase in hydroxypropionic acid metabolites that are indicating the degradation of these amino acids might be attributed to two potential factors: (i) question of quantity: higher dietary intake (ii) question of quality: lower use due to the presence of some limiting amino acid, which causes the rest of the amino acids to be catabolised, which means amino acid imbalances. As mentioned above, the different levels observed in these metabolites between diets can only be explained by the interactions between them, thus amino acids imbalances.

Now, when it is considered that hydroxypropionic acid and hydroxyoctadecadienoic acid may be indicators of amino acid imbalance, we proceed to discuss the results obtained on the levels of these metabolites in the different diets. Diets HHH and LLL showed higher levels of both metabolites than Diets MHL and MMM, and this could be explained as Diet MMM is formulated according current recommendations^18^ and Diet MHL presented the lowest urea levels^46^ (another indication of amino acid deficiency) and the best productive traits in a previous experiment^45^. This is why we can suggest that MMM and MHL diets fitted better to nutritional requirements, with better protein utilisation and a decrease in hydroxypropionic acid and hydroxyoctadecadienoic acid levels. However, Diet LMM (diet with one limiting amino acid) showed similar levels to Diets MMM and MHL. In this case, we provide two possible hypotheses aiming to explain the same levels of these metabolites in “ideal” diets than in a diet with a clearly limiting amino acid. (i) The first aspect addresses variations of these amino acids in the reference diet (Diet MMM). In this case, while Diets LLL and HHH showed differences in the three levels, Diet LMM only differed in the lysine levels. (ii) An alternative explanation could be linked to different dietary intake. In a study where similar diets were used, a reduction in feed intake in the first hour after feed re-administration (-16%) was observed for the diet with lower lysine levels (in this case 4.4 g/kg DM lysine) compared to a diet formulated according to current recommendations^46^. This lower ingestion could be repeated in this experiment and is a possible explanation for the similar levels of these metabolites^83^, which will be proportional to the intake. Regarding validation of the metabolomics analysis, *R*^2^ assesses how well the model fits the observed data, while *Q*^2^ evaluates its predictive power. As outlined in the SIMCA users’ guide, a *Q*^2^ value exceeding 0.5 is admitted for good predictability^84^. Based on our obtained average results for *R*^2^ and *Q*^2^ (0.96 and 0.57, respectively), we can conclude that the models utilised fit well and can be considered as good predictors.

### Both experiments

The experimental design and analysis conducted in this study facilitated the acquisition of plasma metabolic profile after ingestion of diets where the content of the one of three most typically limiting amino acids varied. In view of these results, it can be concluded: (i) The metabolic phenotype undergoes modifications when animals experience a dietary shift in the levels of three amino acids over a short-term period. These alterations in the metabolome allow us to distinctly differentiate the experimental diets, detectable through both targeted and untargeted metabolomics. (ii) The metabolites that were altered by feeding a diet with a limiting lysine level were NEFA, albumin, creatinine, 5-aminopentanamide, lysine phosphoester and enterolactone 3′-glucoronide. These discriminating metabolites seems to be involved in important biological functions linked with protein degradation and microorganism alteration. (iii) Urea, creatinine, albumin, enterolactone 3’-glucoronide, hydroxypropionic acid and hydroxyoctadecadienoic acid are suggested as promising biomarkers for further research into the ideal protein concept.

## Material and methods

### Animal ethics statement

The experimental protocols were approved by the Animal Welfare Ethics Committee of the Universitat Politècnica de València and carried out following the Spanish Royal Decree 53/2013 on the protection of animals used for scientific purposes^85^. We have complied with all relevant ethical regulations for animal use.

### Diets, animals and experimental design

The primary goal of this study was to identify biomarkers that contribute to the implementation of the ideal protein concept in various conditions during the fattening of rabbits. To accomplish this objective, two distinct experiments were implemented:

### Experiment 1

**Fig. 5:**
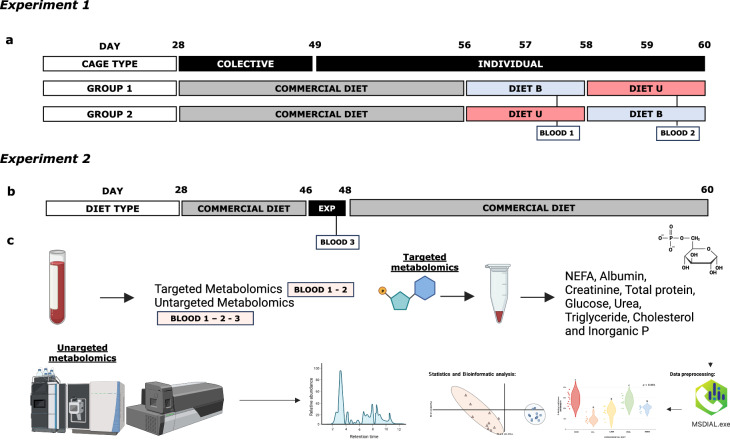
Summary of the experimental design. **a** Representation of the experimental design of the Experiment 1. Twenty-four animals in a replicated latin squares trial trial were used and assigned to one of the two experimental groups (group 1: animals that were first fed with Diet B and then with Diet U and Group 2: opposite order). Blood sampling of selected animals (*n* = 12) was performed at 08:00 h of day 57 and 59, respectively. **b** Experimental design of the Experiment 2. Fifty-three weaned rabbits were used in the study. Animals were initially fed a commercial diet until day 46, when they were assigned to one of the five experimental diets. On day 47, following a 10-h-fasting, blood sampling was conducted at 21:00 h. Subsequently, the animals were once again fed the commercial diet. **c** Representation of the analysis performed with the blood samples (Targeted and Untargeted metabolomics).

A summary of the experimental design is described in Fig. 5a. A total of 24 three-way crossbred growing rabbits (H × LP does inseminated with pooled semen from R bucks; lines H, LP and R from Universitat Politècnica de València, Spain, weaned at 28 day) were used. After weaning, the animals were housed in individual cages (26 × 50 × 31 cm) up to day 49, kept at 10°C to 22°C throughout the experimental period and under a photoperiod of 12 h of light (06:00 to 18:00 h) and 12 h of darkness and fed with a commercial diet (no antibiotics in feed or water were used during the experiment). One day before the first control (56 day of age), the animals were randomly divided into two groups and each group received one of the two experimental diets. Diet with property levels of all amino acids (Diet B) and a diet with a clear deficit of an essential amino acid (Diet U). Group 1: starting with Diet B and Group 2 with Diet U. The following day (57 d of age), feed intake was monitored over 24 h and blood samples were taken from the central ear artery (1 mL in EDTA vials) at 08:00 h. The following day (58 d of age) at 08:00 h, the animals were switched to the other experimental diet and, after a day of adaptation to the new diet, the protocol described for day 57 for feed intake monitoring and blood sampling was repeated on day 59. Blood samples were immediately centrifuged for five min at 700 g, and the plasma was frozen at –20°C until further analysis. The experimental diets were formulated and pelleted from the same basal mixture and introduced just one day before blood sample controls to avoid adaptation mechanisms. Diet B was a balanced diet formulated following the current recommendations for growing rabbits^18^ including 8.1 g of lysine per kg of dry matter (DM) by adding l-lysine HCl (4.7 g per kg). Diet U was an unbalanced diet, which had no l-lysine HCl added, so its lysine content was far from the current recommendations (4.4 g of lysine per kg of DM), ensuring a dietary amino acid imbalance due to a lysine deficit. The ingredients of the basal mixture and the chemical composition of the experimental diets are summarised in Supplementary Table 1.

### Experiment 2

The experimental design of Experiment 2 is shown in Fig. 5b. A total of 53 three-way crossbred growing rabbits (H × LP does inseminated with pooled semen from R bucks; lines H, LP and R from Universitat Politècnica de València, Spain, weaned at 28 days) were used. After weaning, the animals were randomly housed in collective cages (26 × 50 × 31 cm) kept at 10–22 °C throughout the experimental period and under a photoperiod of 12 h of light (06:00 to 18:00 h) and 12 h of darkness and fed *ad libitum* with a commercial diet (with 35 ppm valnemulin and 250 ppm neomycin added). On day 46 of life, the animals were assigned to one of the experimental diets (11, 10, 11, 10, 11, for diet HHH, LLL, LMM, MHL and MMM, respectively). After one day of acclimation (at 08:00 h on day 47), the animals were subjected to 10 h of fasting and refed at 18:00 h, and then, a blood sample was extracted at 21:00 h (3 h after refeeding). This nutritional technique was performed since fasting during the day could optimise, standardise and coordinate feed intake in growing rabbits immediately following refeeding^45^. After the blood extraction, the diet was changed again, providing the same commercial diet until the end of the fattening period (day 60). Blood samples were immediately centrifuged for five min at 700×*g*, and the supernatant plasma was frozen at –20°C until further analysis. Experimental diets were formulated and pelleted from the same basal mixture and were introduced just one day before sampling to avoid adaptation mechanisms (short-term changes). The experimental diets were formulated according to recommendations for crude protein (155 g/kg DM) and digestible energy (9.86 MJ/kg DM) and had the same chemical composition, except for the typically limiting amino acids in growing rabbits; lysine, sulfur amino acids and threonine, whose quantity varied. Medium (M) values were formulated using the current recommendations^18^ and low (L) and high (H) levels were formulated by decreasing or increasing them by 15% in relation with current recommendations, respectively. Experimental diets were obtained by adding synthetic amino acids (l-lysine HCL, dl-methionine and l-threonine) to the same basal mixture, keeping the other ingredients constant. Each diet was named with three letters: first, second and third letters indicate lysine, sulfur amino acids and threonine levels, respectively. The ingredients of the basal mixture and the chemical composition of the experimental diets are summarised in Supplementary Tables 2 and 3.

The selected diets included Diet MMM, aligned with current recommendations (with 8.1, 5.8 and 6.9 g/kg DM of total lysine, sulfur amino acids and threonine, respectively). Diets HHH and LLL maintained the same profile as the recommended diet, but with proportional increases or decreases in amino acid amounts. Diet MHL was chosen for its demonstrated improvement in productive performance in the same animals, signifying a diet correctly tailored to their requirements^46^. Lastly, Diet LMM, known for its lysine deficiency and its impact on plasma urea levels in prior experiments, was also included^45,46,58^.

### Chemical analysis

Chemical analyses of diets (Supplementary Tables 1 and 2) were performed following the Association of Official Agricultural Chemists’ methods^86^ : 934.01 for DM, 942.05 for ash, 976.06 for crude protein and 920.39 with previous acid hydrolysis of samples for ether extract. Starch content was determined by a two-step enzymatic procedure with solubilisation and hydrolysis to maltodextrins with thermostable α-amylase, followed by complete hydrolysis with amyloglucosidase (both enzymes from Sigma-Aldrich, Steinheim, Germany)^87^, and the resulting glucose was measured by the hexokinase/glucose-6 phosphate dehydrogenase/NADP system (R-Biopharm, Darmstadt, Germany). Neutral detergent fibre, acid detergent fibre and acid detergent lignin were analysed sequentially^88^ by method 973.18^86^ and^89^, respectively, with a thermostable α-amylase pre-treatment and expressed exclusive of residual ash, using a nylon filter bag system (Ankom, Macedon, NY, USA). The amino acid content in diets was determined after acid hydrolysis with HCl 6 N at 110°C for 23 h as previously described^90^, using a Waters high-performance liquid chromatography system (Milford, MA, USA). Aminobutyric acid was added as an internal standard after hydrolysis. Amino acids were derivatised with 6-aminoquinolyl-*N*-hydroxysuccinimidyl carbamate and separated with a C-18 reverse-phase column Waters Acc. Tag (150 × 3.9 mm). Methionine and cystine were determined separately as methionine sulfone and cysteic acid, respectively, after performic acid oxidation followed by acid hydrolysis.

Targeted metabolites analysed were non-esterified fatty acids (NEFA), albumin, glucose, creatinine, total protein, urea, triglyceride, cholesterol and inorganic P. Some of them had been studied previously in wild and productive rabbits^30–32^, and are interesting from a nutritional point of view. Blood plasma glucose, albumin, total protein, urea, creatinine, triglyceride, uric acid, cholesterol and inorganic P were determined according to standard procedures (Siemens Diagnostics® Clinical Methods for ADVIA 1800). Finally, NEFAs were determined using the Wako, NEFA C ACS-ACOD assay method. Analyses were performed using an ADVIA 1800 ®Chemistry System Autoanalyzer (Siemens Medical Solutions, Tarrytown, NY 10591, USA).

### LC-MS metabolomics analysis of plasma

#### Chemical solvents and standards for metabolomics analysis

High-performance liquid chromatography (HPLC)-grade solvents and eluents were used for the untargeted metabolomics analysis as follows: HPLC-grade acetonitrile (VWR, West Chester, PA, USA), formic acid (FA, Fluka, Merck KGaA, Darmstadt, Germany) and MilliQ grade water (MilliporeSigma, Burlington, MA, USA). Internal standards included during the sample preparation were glycocholic acid (Glycine-1-13C) and 4-chloro-dl-phenylalanine (Sigma, Merck KGaA, Darmstadt, Germany) and all other standards used for compound identification were purchased from Sigma-Aldrich (Merck KGaA, Darmstadt, Germany) and Cayman Chemical (Ann Arbor, MI, USA).

#### Sample preparation and LC-MS analysis

Plasma from each animal was analysed individually. Plasma was prepared by deproteinisation of 150 µL sample with 450 µL ice-cold acetonitrile (100% acetonitrile [ACN]) containing an internal standard mix of glycocholic acid (glycine-1-^13^C) and p-chlorophenylalanine to a final concentration of 0.01 mg/mL. Samples were prepared in 96-well plates with 1 mL wells. Plates were mixed for 1 min, incubated at 4 °C for 10 min and centrifuged for 25 min at 2250×*g* at 4 °C. Approximately 400 µL of supernatant was transferred to Phenomenex 96-square well filter plates. Vacuum was applied to the plates and the solvent containing plasma metabolites was collected in a collection plate. The filtered supernatant was transferred to two 200 µL 96-well plates (65 µL per well) and plates were vacuum centrifuged to dryness (ca. 2.5 h, 805×*g* at 30  °C). Resuspension of the samples was done in a mix of H_2_O:ACN:FA (95:5:0.1) using the same volume as before evaporation. A protective film was welded onto the plate using a heat sealer, and the plates were centrifuged at 3700 rpm, 4 °C for 25 min before the LC-MS analysis.

The samples were analysed by Ultra High-Performance Liquid Chromatography (UHPLC) using a Nexera X2 LC coupled to an LCMS-9030 Q-TOF MS system (Shimadzu Corporation, Kyoto, Japan) using both positive and negative electrospray ionisation (ESI). Chromatographic separations were performed using an Acquity HSS T3 column (1.7 µm 100 × 2.1 mm, Waters Ltd., Elstree, U.K.). The column temperature was set to 40 °C, the samples were maintained at 10 °C and 3 µl aliquots were injected onto the column. The chromatographic system used a binary gradient of Solvent A (water with 0.1% formic acid) and Solvent B (acetonitrile with 0.1% formic acid) with a flow rate of 0.4 mL/min. A linear gradient was used from 5% B to 100% B over 12 min, and 1 min hold at 100% before returning to the initial conditions of 5% B for 3 min for column re-equilibration. This resulted in a total analysis time per sample of 16 min. MS detection was performed using a data-independent acquisition (DIA) method for MS and MS/MS analyses. The method acquired a single time-of-flight (TOF) MS scan (m/z 50–900) followed by 33 MS/MS mass scans over a mass range of m/z 40-900; each MS/MS mass scan had a precursor isolation width of 25.2 Da and a collision energy spread of 10–30 V, resulting in a cycle time of 0.9 s. This allowed collection of fragmentation data of all masses in the spectra across the entire LC gradient. The following MS parameters were used: ion-source temperature, 300 °C; heated capillary temperature, 250 °C; heat block temperature, 400 °C; electrospray voltage 4.5 kV (ESI + ) or −3.5 kV (ESI-); electrospray nebulisation gas flow, 3 L/min; drying gas flow, 10 L/min; detector voltage, 2.02 kV. Mass calibration was performed externally using a sodium iodide solution (400 ppm in methanol) from m/z 50–1000. Data acquisition was performed using LabSolutions software version 5.114 (Shimadzu Corporation, Kyoto, Japan).

#### Sample quality control and metabolomics data pre-processing

The quality of the chromatographic runs, the UPLC system stability and the accuracy of sample preparation were monitored using quality control samples (QCs). Plasma QCs were prepared by pooling an aliquot of all samples and subjecting this pooled sample to the same sample preparation protocol as the samples. The QCs were injected multiple times throughout the analysis as well as at the beginning and end of the analysis and used in the data pre-processing for signal drift correction. Blanks were injected during the chromatographic analysis to monitor any external contaminants from solvents, eluents and carry-over effects. The sample order was randomised for the chromatographic analysis to eliminate biases in the results and to ensure that each sample group was affected equally.

MS-DIAL software^91^ was used to perform peak detection, alignment and gap filling for the data files. The MS-DIAL generated data matrix was exported to Excel and filtered to eliminate peaks present in blanks, and retention time was truncated to contain only portions containing chromatographic peaks, while masses higher than 700 m/z were discarded.

Initial principal component analysis (PCA) was performed using LatentiX 2.12 (LATENTIX Aps., Gilleleje, Denmark) to check the quality of the data set and eliminate potential outliers. Partial least-squares discriminant analysis (PLS-DA) models were built to determine the metabolites responsible for the differences between experimental diets. Validation of the models was performed using repeated random subsampling validation. Models were assessed using the explained variation in Y, plots depicted actual and predicted values, and the proportion of variation explained (R2). Variables for identification were selected using variable importance in projection (VIP) scores. To validate the obtained PLS-DA models, a cross-validation was performed using 5 as the maximum number of components to search, along with the 5-fold CV method. For each generated PLS model, both *R*^2^ and Q2 were calculated.

#### Metabolite identification

Metabolites were identified based on queries in the Human Metabolome Database (http://www.hmdb.ca) online database to obtain possible chemical structures using accurate mass and mass spectrometric fragmentation patterns.

### Metabolites statistical analysis

The identified metabolites were statistically analysed with the GLM procedure of SAS (2002). The model for Experiment 1, included as fixed effects the animal (each of the selected animals becomes a block), the control day (57 and 59 days of age) and the experimental diet (Diet B and Diet U). The model for Experiment 2 included as fixed effects the animal (each of the selected animals becomes a block) and the experimental diet (Diet HHH, Diet LLL, Diet LMM, Diet MHL and Diet MMM). Least square means were obtained with their standard errors and compared using *t*-test, defining significance level at *p* < 0.05. Orthogonal contrasts between experimental diets were also performed (*t*-test).

### Reporting summary

Further information on research design is available in the Nature Portfolio Reporting Summary linked to this article.

### Supplementary information

Supplemetary Tables 1-3

Description of additional supplementary files.

Supplementary Data 1

Reporting summary

## Data Availability

All data generated or analysed during this study are included in this published article (and its Supplementary Information files).
