# Supplementary material for: Biomarkers for ideal protein: rabbit diet metabolomics varying key amino acids
Source: Commun Biol. 2024 Jun 10;7:712. doi: 10.1038/s42003-024-06322-2 (PMC11164918; doi:10.1038/s42003-024-06322-2)
Supplement: Supplementary file 1 — Supplemetary Tables 1-3 [file 42003_2024_6322_MOESM1_ESM.docx]

| **Supplementary Table 1:** Ingredients and chemical composition of experimental diets used in Experiment 1. | | | | | |
| --- | --- | --- | --- | --- | --- |
| **Ingredients (g/kg)** | **BM^2^** | | **Chemical composition (g /kg DM)** | **B^3^** | **U^3^** |
| Wheat grain | 288 | | Dry matter | 912 | |
| Sunflower meal | 165 | | Ash | 88 | |
| Soybean oil | 40 | | Crude protein | 158 | |
| Cereal straw | 83 | | Ether extract | 61 | |
| Alfalfa hay | 360 | | Starch | 183 | |
| Defatted grape seed | 45 | | Neutral detergent fiber (NDF) | 366 | |
| **L-lysine HCl** | **0** | | Acid detergent fiber (FAD) | 216 | |
| DL-Methionine | 1.55 | | Acid detergent lignin (ADL) | 57 | |
| L-Threonine | 2.2 | | Digestible energy (Mj/kg/DM)^4^ | 10.7 | |
| Dicalcium phosphate | 3.6 | | Digestible protein^4^ | 109 | |
| L-Arginine | 1.45 | | Amino acid composition: |  | |
| Sodium chloride | 5.2 | | Aspartic acid | 13.4 | |
| Vitamine/mineral^1^ | 5 | | Serine | 60.1 | |
|  |  | | Glutamic acid | 20.9 | |
|  |  | | Glycine | 70.8 | |
|  |  |  | Histidine | 2.51 | |
|  |  |  | Arginine | 8.10 | |
|  |  |  | Threonine | 6.88 | |
|  |  |  | Alanine | 5.13 | |
|  |  |  | Proline | 8.57 | |
|  |  |  | Cystine | 1.56 | |
|  |  |  | Tyrosine | 2.67 | |
|  |  |  | Valine | 6.73 | |
|  |  |  | Methionine | 3.23 | |
|  |  |  | Isoleucine | 5.13 | |
|  |  |  | **Lysine** | **8.10** | **4.40** |
|  |  |  | Leucine | 8.80 | |
|  |  |  | Phenylalanine | 6.47 | |
| 1 Contains per kg of feed: vitamin A: 8375 IU; vitamin D3: 750 IU; vitamin E: 20 mg; Vitamin K3: 1 mg; vitamin B1: 1 mg; vitamin B2: 2 mg; vitamin B6: 1 mg; nicotinic acid: 20 mg; choline chloride: 250 mg; magnesium: 290 mg; manganese: 20 mg; zinc: 60 mg; iodine: 1.25 mg; iron: 26 mg; copper: 10 mg; cobalt: 0.7 mg; butyl hydroxylanysole and ethoxiquin mixture: 4 mg. 2 BM: Basal Mixture 3 B: Balanced Diet added with 4.7 g of L-Lysine HCl of the basal mixture; U: Unbalanced Diet, same composition than basal mixture 4 Calculated values from FEDNA (2010). | | | | | |

| **Supplementary Table 2:** Ingredients and chemical composition of basal mixture of the experimental diets used in Experiment 2. | | | | |  |
| --- | --- | --- | --- | --- | --- |
| **Ingredients** | **g/kg** | | **Chemical composition** | **g/Kg DM** | |
| Wheat gran | 300 | | Dry matter^2^ | 907 | |
| DDGS corn | 50 | | Ash^2^ | 104 | |
| Bakery by-product | 30 | | Crude protein^2^ | 155 | |
| Sunflower meal | 36 | | Crude fat^2^ | 29.7 | |
| Alfalfa meal | 334 | | Neutral detergent fiber (NDF) ^2^ | 455 | |
| Beet pulp | 80 | | Acid detergent fiber (FAD) ^2^ | 29.7 | |
| Straw | 136 | | Acid detergent lignin (ADL) ^2^ | 455 | |
| Beet molasses | 13.9 | | Digestible energy (Mj/kgDM)^2^ | 9.86 | |
| L-Arginine | 3.1 | | Amino acid composition^3^: |  | |
| L-Histidine | 1.5 | | Aspartic acid | 12.95 | |
| Calcium carbonate | 6.5 | | Serine | 5.74 | |
| Sodium clorhide | 4 | | Glutamic acid | 22.96 | |
| Vitamine/mineral^1^ | 5 | | Glycine | 6.53 | |
|  |  | | Histidine | 3.57 | |
|  |  | | Arginine | 9.28 | |
|  |  |  | Threonine | VAR^4^ | |
|  |  |  | Alanine | 6.69 | |
|  |  |  | Proline | 8.04 | |
|  |  |  | Cystine | 2.37 | |
|  |  |  | Tyrosine | 2.90 | |
|  |  |  | Valine | 7.03 | |
|  |  |  | Methionine | VAR^4^ | |
|  |  |  | Isoleucine | 5.07 | |
|  |  |  | Lysine | VAR^4^ | |
|  |  |  | Leucine | 9.70 | |
|  |  |  | Phenylalanine | 5.58 | |
| 1 Contents per kg of feed: vitamin A: 8375 IU; vitamin D3: 750 IU; vitamin E: 20 mg; Vitamin K3: 1 mg; vitamin B1: 1 mg; vitamin B2: 2 mg; vitamin B6: 1 mg; nicotinic acid: 20 mg; choline chloride: 250 mg; magnesium: 290 mg; manganese: 20 mg; zinc: 60 mg; iodine: 1.25 mg; iron: 26 mg; copper: 10 mg; cobalt: 0.7 mg; butyl hydroxyanisole and ethoxyquin mixture: 4 mg. 2 Provided by the manufacturer (NANTA, Valencia, Spain). 3 Analysed. 4 Levels of these amino acids vary depending on experimental diets. d Variable amino acid levels in the experimental diets (lysine, sulphur amino acids and threonine). | | | | | |
|  | | | | | |

| **Supplementary Table 3:** Variable amino acid (AA) levels in the experimental diets. | | | |
| --- | --- | --- | --- |
|  | Lysine level | Sulphur AA (Cysteine + Methionine) | Threonine |
| Diet HHH | 9.4 | 6.6 (2.37 + 4.23) | 7.8 |
| Diet LLL | 6.7 | 4.9 (2.37 + 2.53) | 5.7 |
| Diet LMM | 6.7 | 5.8 (2.37 + 3.43) | 6.9 |
| Diet MHL | 8.1 | 6.6 (2.37 + 4.23) | 5.7 |
| Diet MMM | 8.1 | 5.8 (2.37 + 3.43) | 6.9 |
