# Supplementary material for: Biomarkers for ideal protein: rabbit diet metabolomics varying key amino acids
Source: Commun Biol. 2024 Jun 10;7:712. doi: 10.1038/s42003-024-06322-2 (PMC11164918; doi:10.1038/s42003-024-06322-2)
Supplement: Supplementary file 2 — Description of additional supplementary files. [file 42003_2024_6322_MOESM2_ESM.docx]

Description of Additional Supplementary Files

**File name:** Supplementary Table 1

**Description:** Ingredients and chemical composition of experimental diets used in Experiment 1.

**File name:** Supplementary Table 2

**Description:** Ingredients and chemical composition of basal mixture of the experimental diets used in Experiment 2.

**File name:** Supplementary Table 3

**Description:** Variable amino acid (AA) levels in the experimental diets.

**File name:** Supplementary Data 1

**Description:** Source data for all the figures and plots in the manuscript.
